# Proteomic Analysis of Blood Exosomes from Healthy Females and Breast Cancer Patients Reveals an Association between Different Exosomal Bioactivity on Non-tumorigenic Epithelial Cell and Breast Cancer Cell Migration in Vitro

**DOI:** 10.3390/biom10040495

**Published:** 2020-03-25

**Authors:** Oleg Tutanov, Evgeniya Orlova, Ksenia Proskura, Alina Grigor’eva, Natalia Yunusova, Yuri Tsentalovich, Antonina Alexandrova, Svetlana Tamkovich

**Affiliations:** 1 Laboratory of Molecular Medicine, Institute of Chemical Biology and Fundamental Medicine, Siberian Branch of Russian Academy of Sciences, 630090 Novosibirsk, Russia; ostutanov@gmail.com (O.T.); ksen-84@list.ru (K.P.); feabelit@mail.ru (A.G.); 2 Laboratory of Carcinogenesis Mechanisms, “N.N. Blokhin Cancer Research Center” of the Ministry of Health of the Russian Federation, 115478 Moscow, Russia; evg19111976@yandex.ru (E.O.); tonya_alex@yahoo.com (A.A.); 3 Department of Mammology, Novosibirsk Regional Clinical Oncological Dispensary, 630108 Novosibirsk, Russia; 4 Laboratory of Tumor Biochemistry, Cancer Research Institute, Тomsk National Research Medical Center, Russian Academy of Science, 634028 Tomsk, Russia; bochkarevanv@oncology.tomsk.ru; 5 Department of Biochemistry and Molecular Biology, Siberian State Medical University, 634050 Tomsk, Russia; 6 Laboratory of Proteomics and Metabolomics, International Tomography Center SB RAS, 630090 Novosibirsk, Russia; yura@tomo.nsc.ru; 7 Department of Molecular Biology and Biotechnology, Novosibirsk State University, 630090 Novosibirsk, Russia

**Keywords:** exosomes, migration, mass-spectrometry, breast cancer

## Abstract

Exosomes are important intercellular communication vehicles, secreted into body fluids by multiple cell types, including tumor cells. They contribute to the metastatic progression of tumor cells through paracrine signalling. It has been recently discovered that blood circulating exosomes contain distinguishable fractions of free and cell-surface-associated vesicles. We evaluated the influence of protein cargoes from exosomes from plasma, and exosomes from the total blood of healthy females (HFs) and breast cancer patients (BCPs), on cell motility. We conducted a mass spectrometric analysis of exosomal contents isolated from samples using ultrafiltration and ultracentrifugation approaches and verified their nature using transmission electron microscopy, nanoparticle tracking analysis and flow cytometry. We observed that malignant neoplasm-associated proteins in exosomes from BCP total blood were detected more often than in plasma (66% vs. 59%). FunRich analysis to assess Gene Ontology (GO) enrichment revealed that proteins with catalytic activities, transporter functions and protein metabolism activities were increased in exosomes from BCP blood. Finally, GO analysis revealed that proteomic profiles of exosomes from HF total blood were enriched with proteins inhibiting cell migration and invasion, which explains the low stimulating activity of exosomes from HF total blood on SKBR-3 cancer cell migration velocity. This allows exosomes to act as intermediaries providing intercellular communications through horizontal transfer of RNA and functionally active proteins, potentially affecting the development of both primary neoplasms and distant metastases.

## 1. Introduction

Exosomes are small (30–100 nm) membrane vesicles released into the extracellular environment after the fusion of multivesicular bodies with the plasma membrane [1]. Exosome release occurs under normal physiological conditions and abnormal conditions such as cancer and other diseases. Secreted cancer cell exosomes are carried through the blood and lymph circulation and may be detected far away from parental cells [2]. These vesicular structures are highly stable and are small enough to penetrate into and interact with different tissues. Exosomes are involved in key physiological processes such as cell-to-cell communications [3], proliferation [4], migration [5], invasion [6], angiogenesis [7], and tumor growth [8] through the horizontal transfer of RNA and proteins.

Exosomal surface-exposed receptors and ligands are responsible for their bio-distribution and binding to target cells or extracellular matrices [9,10]. When exosomes circulate in the blood, they are in contact with blood cells, and after such interactions with cell plasma membranes, some do not undergo immediate membrane fusion/internalization but remain at the cell surface as cell-associated vesicles for some time. Indeed, it has been recently discovered that blood circulating exosomes contain distinguishable fractions of free and cell-surface-associated vesicles [11,12]. The role of blood cell-surface-associated exosomes has not been fully elucidated. Recently, we demonstrated that miRNA and proteins from blood cell-bound exosomes represented valuable sources of materials for cancer diagnostics [11,12].

In the present work, we explored the biological activity of cell-free and blood cell-surface-associated exosomes, materials that were derived from a previous study [12]. We showed that protein cargoes in exosomes from plasma and total blood of healthy females (HFs) and breast cancer patients (BCPs) correlated with redistribution between cell-free and cell-associated fractions of exosomes, and with alterations in the motility characteristics of certain cells.

## 2. Material and Methods

### 2.1. Ethics Statement

Blood samples from previously untreated luminal BCPs (n = 23, age 31–79 years, median age 58) were obtained from Novosibirsk Regional Oncology Dispensary (Table 1).

Blood samples from HFs (n = 21, age 38–65 years, median age 56), collected for blood transfusion, were obtained from Novosibirsk Central Clinical Hospital. HFs did not have any female-related disorders (mastopathy, endometriosis, etc.) or malignant diseases. Written informed consent was obtained from every participant and the Ethical Committees of the Dispensary, Hospital and Institute approved the studies. Human samples were obtained according to the principles expressed in the Declaration of Helsinki.

### 2.2. Exosome Isolation

Venous blood (9 mL) was collected in K_3_EDTA spray-coated vacutainers (Improvacuter, Guangzhou, China, cat. no. 694091210), immediately mixed using a rotary mixer, placed at + 4 °C and processed within an hour after blood taking.

The blood sample was divided into two equal parts. One part was used for the isolation of plasma exosomes, and the second for the isolation of total blood exosomes. To isolate the plasma exosomes, blood cells were pelleted by centrifugation at 290 g for 20 min, blood plasma was transferred to a new tube and centrifuged a second time at 1200 g for 20 min. To remove cell debris, plasma samples were centrifuged at 17,000 g at 4 °C for 20 min. 

Plasma supernatants were diluted in PBS (P4417, Sigma, Saint Louis, Missouri, USA) with 5 mM EDTA in a 1:5 ratio, passed through a 100 nm pore-size filter (Minisart high flow, 16553-K, Sartorius, Göttingen, Germany), and the filtrates centrifuged for 90 min at 100,000 g (4 °C). Pellets were suspended in 12 mL PBS and again centrifuged for 90 min at 100,000 g (4 °C). This washing stage was performed three times. Then supernatants were removed and pellets resuspended in 150 μL PBS.

To isolate total blood exosomes, a previous protocol was used [12]. Briefly, equal volumes of elution buffer (BioSilica Ltd, Novosibirsk, Russia) were added to blood samples, and incubated on a rotary mixer (10 rpm) for 4 min, at room temperature. Total blood exosomes were isolated using the same procedures as for plasma exosome isolation.

To study the impact of exosomes on cell migration, half the exosome samples from different individuals were mixed, to generate two samples (plasma exosomes and total blood exosomes) from HFs and two samples (plasma exosomes and total blood exosomes) from BCPs. Individual and mixed samples were frozen in liquid nitrogen and stored in aliquots at −80 °C until required.

### 2.3. Electron Microscopy of Exosomes

For negative staining, a drop of exosomes was incubated for 1 min on a copper grid covered with formvar film, stabilized by carbon. Then, grids were exposed for 5–10 s on a drop of 0.5% uranyl acetate or 2% phosphotungstic acid. The grids holding the adsorbed exosomes, or ultrathin sections, were examined on a transmission electron microscope (TEM) (JEM 1400 (Jeol, Tokyo, Japan) with a digital camera Veleta (EMSIS, Münster, Germany)). Exosome measurements were made directly on the camera screen using iTEM (EMSIS, Münster, Germany) software.

### 2.4. Nanoparticle Tracking Analysis

Exosome quantities were analyzed using a nanoparticle tracking analysis (NTA) system, NanoSight NS300 (Malvern, Surrey, UK). Depending on the concentration of the particles, the samples were diluted 50–100 fold in 0.1 μm filtered PBS to obtain optimal conditions for NTA concentration measurements. Each sample was measured in triplicate, with a camera setting of 15, an acquisition time of 30 s and a detection threshold setting of 5. At least 200 completed tracks were analyzed per video. NTA analytical software version 2.3 was used for data analysis and capture.

### 2.5. Protein Quantification

To estimate protein concentrations, 7.5 μL of an exosome suspension was mixed with lysis buffer (0.25 M Tris-HCl, 8% SDS, 0.2 М DTT, pH 6.8), incubated on ice (10 min), boiled (95 °C for 10 min) and cooled. After centrifugation (12,000 g, 10 min), the protein concentration was measured using a fluorometric protein assay (NanoOrange® Protein Quantitation Kit, Molecular Probes, Waltham, USA) according to the manufacturer’s instructions.

### 2.6. Flow Cytometry Analysis 

For exosome immunoprecipitation and their subsequent analysis by fluorescence-activated cell sorting (FACS), 4 μm diameter aldehyde/sulfate latex beads (Interfacial Dynamics, Portland, Oregon, USA) were incubated with purified anti-CD9 or anti-CD24 (BD Biosciences, San Jose, CA, USA) or anti-ADAM-10 (Abcam, Cambridge, UK) antibodies at 22 °C overnight, with gentle agitation as previously described [13]. For FACS analysis, 30 μg exosomes were incubated with 3 × 10^5^ anti-CD9, anti-CD24 or anti-ADAM-10 beads in 150 μL of PBS at 4 °C overnight, with gentle agitation. The reaction was stopped by incubation with 0.2 M glycine for 30 min. The exosome-bead complexes were washed twice in FACS buffer (3% exosome depleted fetal bovine serum (FBS, Gibco, Gaithersburg, Maryland, USA) in PBS). The bead-bound exosomes were then incubated with human IgG (BD Biosciences, San Jose, CA, USA) at 4°C for 30 min, with subsequent washing in FACS buffer, and incubation with fluorescein-conjugated anti-CD9, anti-CD24, anti-CD63, anti-CD81 or isotype antibodies (BD Biosciences, San Jose, CA, USA) for 40 min at room temperature, with gentle agitation. All complexes were washed twice, suspended in 300 μL of FACS buffer and analyzed by flow cytometry on the FACS Canto II (BD Biosciences, San Jose, CA, USA), using FACS Diva 6.1 Software. The median fluorescence intensity (MFI) of stained exosomes was captured and analyzed and compared to the isotype control.

### 2.7. Cell Lines

MCF10A non-tumorigenic epithelial cells (ATCC® CRL-10317™), were cultured in Dulbecco’s Modified Eagle Medium/Nutrient Mixture F-12 (DMEM/F12) supplemented with 5% horse serum, GlutaMAX-I (10 μL/mL), epidermal growth factor (EGF) (20 ng/mL), hydrocortisone (0.5 μg/mL), cholera toxin (100 ng/mL), insulin (10 μg/mL), NaHCO_3_ (32.5 μg/mL), penicillin (100 U/mL), and streptomycin (100 μg/mL). SKBR-3 human breast cancer cells (ATCC® HTB-30™), were cultured in Dulbecco’s Modified Eagle’s Medium (DMEM) supplemented with 10% FBS, penicillin (100 U/mL) and streptomycin (100 μg/mL). Some assays were performed in the absence of serum media, and the presence or absence of epidermal growth factor.

### 2.8. Cell Migration Assay

For 2D migration assays, cells were plated onto an 8-well slide (NuncTM Lab-TekTM, ThermoScientifiс, Loughborough, UK), at a density of 12 × 10^3^ and 13.5 × 10^3^ cells/well, for MCF10A and SK-BR-3, respectively. For MCF10A cells, the slides were coated with 1 mg/mL bovine fibronectin (Sigma-Aldrich, Darmstadt, Germany) before experiments. All 8-well slides were then incubated at 37 °C in a humidified atmosphere at 5% CO_2_ for 24 h (SKBR-3) or 48 h (MCF10A). The culture medium was then replaced by DMEM (SKBR-3) or DMEM/F12 (MCF10A), without serum and EGF for 5 h. After this period, the medium was changed to F12 medium with 15 mM HEPES, with or without serum/EGF/exosomes. We added 1–2 × 10^8^ exosomes per well, which was similar to exosome concentrations in plasma, and based on our and others’ previous findings [11]. Slides were placed inside a 37 °C incubation chamber of a Nikon Eclipse-Ti microscope, equipped with an ORCA-ER Hamamatsu camera controlled by NIS-Elements AR2.30 software (Nikon, Tokyo, Japan). Images were acquired with a Plan-Neofluar ×10 and ×20 objectives every 5 min, for 15–20 h. To estimate cell motility, we analyzed the number of motile cells under each condition (a migrated cell was defined if it moved a distance longer than its radius), and the velocity of moving cells, based on tracking cell migration using MTrackJpluging in ImageJ. Two-three fields of view were recorded for each condition, in three independent experiments.

### 2.9. Mass Spectrometry Analysis

Individual exosome samples were separated based on their molecular weight using SDS disc electrophoresis. The gels were stained by Coomassie Brilliant Blue R250 (Sigma, Darmstadt, Germany). The PAAG fragments containing proteins under study were treated using the modified Rosenfeld method [14]. Briefly, PAAG fragments with proteins were washed from Coomassie R250 and SDS with an aqueous solution containing 50% acetonitrile and 0.1% trifluoroacetic acid. Proteins absorbed in the gel were reduced with 45 mM DTT in 0.2 M ammonium bicarbonate at 60 °С for 30 min, followed by protein alkylation with 100 mM iodoacetamide in 0.2 M ammonium bicarbonate, at room temperature for 30 min. The gel fragments were dehydrated in 100% acetonitrile. A 0.2 mM trypsin solution (modified by reductive methylation) (Sigma, T6567, Darmstadt, Germany), in a mixture with 0.1 M ammonium bicarbonate and 5 μM CaCl_2_, was added to each gel fragment and incubated for 30 min at room temperature. Then, peptide extraction buffer (60 μL) was added to the gel fragments, and samples were incubated for 16–18 h at 37 °С. The peptide fragments of proteins extracted from the gel were concentrated and desalted using C18 ZipTips micro-columns (Millipore, Darmstadt, Germany) according to the manufacturer’s instructions. The peptide mixture was eluted from the micro-column, on a target of the device plate with the saturated matrix solution.

Mass-spectra were registered at the Center of Collective Use «Mass spectrometric investigations» SB RAS on an Ultraflex III MALDI-TOF/TOF mass spectrometer (BrukerDaltonics, Bremen, Germany) in positive mode, with the range 700–3000 Da, and with 2,5-dihydroxybenzoic acid as a matrix. Proteins were identified by searching for appropriate candidates in annotated NCBI and SwissProt databases using Mascot software (Matrix Science Ltd., London, UK, www.matrixscience.com/search_form_select.html). The following parameters were used for searches: the acceptable mass deviation of the charged peptide (50 ppm)—0.05Da; the acceptable number of missed cleavage sites—2; carbamidomethylation of cysteine residues was chosen as a fixed modification and the presence of oxidized methionine residues was chosen as a variable modification; identification reliability not lower than 95%.

### 2.10. Bioinformatics and Gene Ontology (GO) Analysis of Proteomic Profiles

Venn diagrams, illustrating shared proteins from exosome samples from plasma and total blood, as well as GO enrichment analysis of the exosomal proteome, were performed using a FunRich version 3.1.3 software package [15]. GO profiling of exosomal proteins involved in cell migration and motility was performed using the QuickGO annotation terms (lists of obtained proteins were searched against GO terms cell motility (GO:0048870), cell migration (GO:0016477) and negative regulation of cell motility (GO:2000146)) [16]. Profiling of the differently expressed exosomal proteins during the development of various malignant diseases was performed using the dbDEPC 3.0 database [17]. The involvement of exosomal proteins in cancer invasion was routinely analyzed by searching the PubMed database on relevant publications for each protein.

### 2.11. Statistical Analysis

Statistical calculations were performed using Statistica 6.0 software and GraphPad PRISM 5 software (GraphPad Software, La Jolla, CA, USA). All data were expressed either as the median with interquartile ranges or as means with standard errors. To evaluate differences, the Mann–Whitney U-test was performed. *p* values < 0.05 were considered statistically significant. Cell motility data represented at least four independent experiments for the SKBR-3 cell line, and two independent experiments for the MCF10A cell line.

## 3. Results

### 3.1. Characterization of Plasma and Total Blood Vesicles as Exosomes

**Figure 1:**
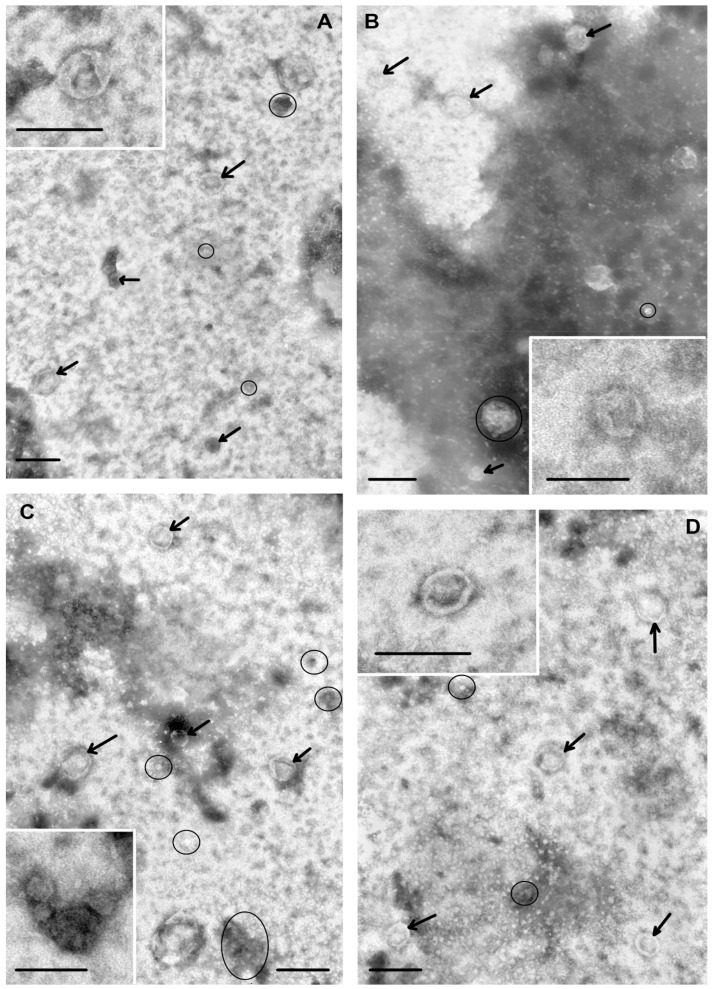
Total view of exosome preparation obtained from: blood plasma of HFs (**A**), blood plasma of BCPs (**B**), total blood of HFs (**C**), total blood of BCPs (**D**). Inserts show exosomes. Arrows indicate exosomes, ellipses—«non-vesicles». Scale bars correspond to 100 nm. Electron microscopy, negative staining by phosphotungstate acid.

The morphology of single plasma vesicles and total blood vesicles was examined by TEM to reveal spherical vesicles of 40–100 nm in diameter, with a bilayer membrane (Figure 1). Vesicles with damaged membranes did not exceed 10%, and the portion of microvesicles (with the size smaller than 30 nm), was no more than 15%. NTA analysis revealed that vesicle sizes were within the 31–226 nm range, with a median hydrodynamic radius of 96–131 nm (Table 2). Although both NTA and TEM demonstrated similar extracellular vesicles characteristics in terms of size distribution, in general, the size values measured by TEM can be lower than those measured by NTA, as recently described [18,19,20].

The presence of exosomal markers was confirmed by flow cytometry. Two types of monoclonal antibodies were used to evaluate exosome maturity: antibodies to the tetraspanin family of receptors, CD9, CD63 and CD81, which are essential structural components of exosome membranes that mediate exosome adhesion to recipient cell surfaces [21], and the antibody specifically binding to the CD24 receptor, which is a marker of actively dividing and differentiating cells, including epithelium [22]. Vesicles isolated from plasma and total blood expressed CD63, CD9, CD24 and CD81 (Table 3); thus, they were classified as exosomes. Collectively, these data demonstrate that vesicles isolated from plasma and total blood demonstrate all characteristics of exosomes.

### 3.2. Quantitative and Sub-Population Analysis of Exosomes from Plasma and Total Blood

NTA assessed the size and concentration of pooled exosomes in our samples. NTA was performed in triplicate for each sample, and the reproducibility of counting between runs was approximately 15%. Plasma exosome sizes from HF blood and triple-negative breast cancer type patient’s blood were lower than total blood exosomes, except for luminal subtype BCPs (Table 2). To characterize the proportion of cell-free and blood cell-associated exosomes, the concentrations of plasma exosomes and total blood exosomes were estimated (Table 2). The data indicated that 31% of blood exosomes circulated in HF plasma, 39% in triple-negative subtype BCP plasma, and 96% in luminal subtype BCP plasma. Thus, the proportion of blood cell-associated exosomes in breast cancer was decreased, in comparison with the healthy state.

Various exosome types circulate in the blood of healthy donors and patients with different cancers, and surface proteins mainly represented by CD9, CD24, CD63 and CD81 are considered as universal markers of blood exosomes [23]. Using combinations of unconjugated and conjugated antibodies, we characterized subpopulations of exosomes from plasma and total blood and found that subpopulation compositions of exosomes from plasma and total blood of HFs and BCPs were similar: CD24/CD9 > CD9/CD81 > CD24/CD63 ≈ CD9/CD63 (Table 3). Thus, in both cell-free and blood cell-associated samples of HFs and BCPs, exosomes expressing CD9 and CD24 receptors were most frequently detected.

### 3.3. Exosomes from Plasma and Total Blood Increase Cell Migration Activities

The effects of exosomes from plasma and total blood from HFs and BCPs, on cell migration, were tested in MCF10A and SKBR-3 cells. MCF10A is a spontaneously immortalized, non-malignant breast cell line from a patient with benign fibrocystic disease, and is the founder cell line of a more progressively aggressive family of breast cancer lines [24]. These cells, therefore, act as a useful control cell model to assess the oncogenic potential of blood exosomes from cancer patients.

**Figure 2:**
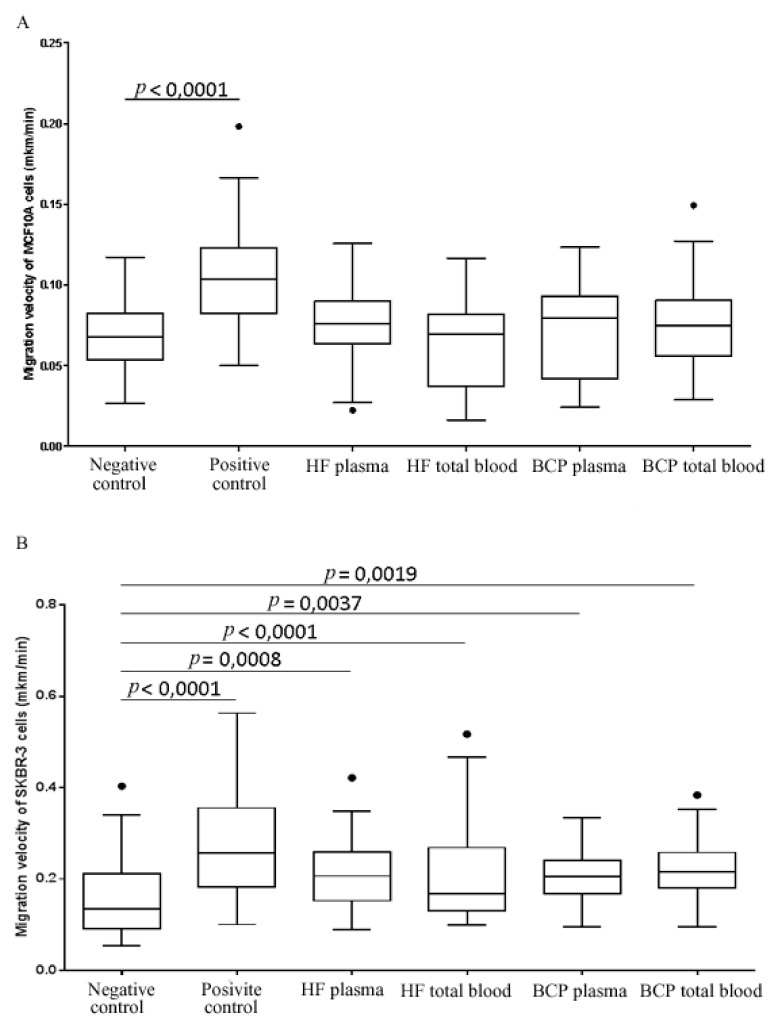
The velocity of migrating MCF10A (**A**) and SKBR-3 (**B**) cells under specified conditions. Results are presented as Tukey box plots. Median (-) velocity with 25–75% (□) and non-outlier range bars are indicated.

Non-tumorigenic epithelial MCF10A cells on 2D surfaces are located singly or combined into cell islands of various sizes. Under serum-free and EGF-free conditions, the cells were practically immobile and established good cell-to-cell contacts. The addition of horse serum and/or EGF to these cells causes mobility stimulation (Table 4). Migrated cell numbers and their velocities increased significantly for both individual MCF10A cells and cells within islands (Figure 2A, Table 4, Figure S1A). The addition of exosomes from HF plasma and from plasma and total BCP blood led to a significant increase in the motile cell numbers in the absence of serum and EGF (Table 4); similarly, the movement of several whole-cell islands toward each other was also observed. Exosomes from HF total blood did not cause this effect: the migration was observed only for several cells at the periphery of an island. It should be noted that exosome addition does not affect the migration velocity of MCF10A cells (Figure 2A, Figure S1A).

Negative controls include cells without HS and EGF for MCF10A and FCS for SKBR-3. Positive controls include cells with HS and with EGF for MCF10A and with FCS for SKBR-3. Each experiment was repeated 4 to 7 times. The significance of cell stimulation was evaluated by comparison with the corresponding negative control. NS – non-significant.

SKBR-3 tumor cells in DMEM supplemented with 10% FCS have fibroblast-like shapes. These cells, even in dense culture and inside groups move actively. The maximal increase in motile cell numbers and the highest migration rates were observed in cells supplemented with 10% FCS, without exosomes (Table 4). Cell washing with DMEM without serum led to a significant weakening in cell migration, suggesting FCS had the greatest impact on SKBR-3 cell motility. The addition of exosomes from HFs and BCPs to control cells without calf serum contributed to the increase in the number of single tumor cells capable of promoting the migration by 2.5-3 times, but no statistically significant differences have been observed (Table 4, Figure S1B). Furthermore, we found that any exosomes (from plasma and total blood of HFs or BCPs), significantly stimulated the velocity of movement of cancer cells. The lower effect on the increase in cell velocity had the addition of total blood exosomes from HFs (Figure 2B). In particular, the median migration velocity of SKBR-3 cells after adding exosomes from HF plasma, BCP plasma and BCP total blood were; 0.2062 μm/min, 0.2045 μm/min and 0.2162 μm/min, respectively. After adding exosomes from HF total blood, the rate was 0.1673 μm/min (Figure 2B, Figure S1B).

### 3.4. Mass-spectrometry Analysis of the Exosomal Proteome Reveals Proteins Associated with Cell Motility and Invasiveness

After 1D SDS-PAGE exosomal protein separation (Figure S2), the whole lane was cut into 25 bands of about 2 mm each. Followed by in-gel trypsin digestion, the peptides were extracted from each band and then loaded to MALDI-TOF/TOF mass spectrometer for protein identification, respectively.

**Figure 3:**
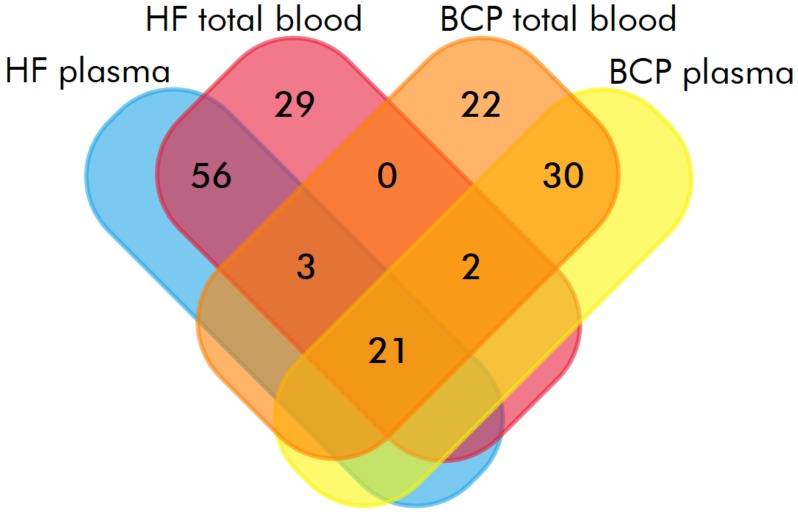
Venn–Euler diagram of proteins in exosomes from HF and BCP blood.

In total, 111 and 146 proteins were identified with high reliability (P < 0.05) by MALDI-TOF mass-spectrometry, in exosomes from HF and BCP blood, respectively. Of these, 34 were common between groups (Figure 3, Table 5). The proportions of proteins detected only in the total blood exosomes of HFs and BCPs were 24% and 42%, respectively (Table 6 and Table 7). Approximately 45% of identified exosomal proteins were previously discovered in other studies, using mass spectrometry and annotated in the Exocarta database (www.exocarta.org) (Tables S1 and S2).

**Figure 4:**
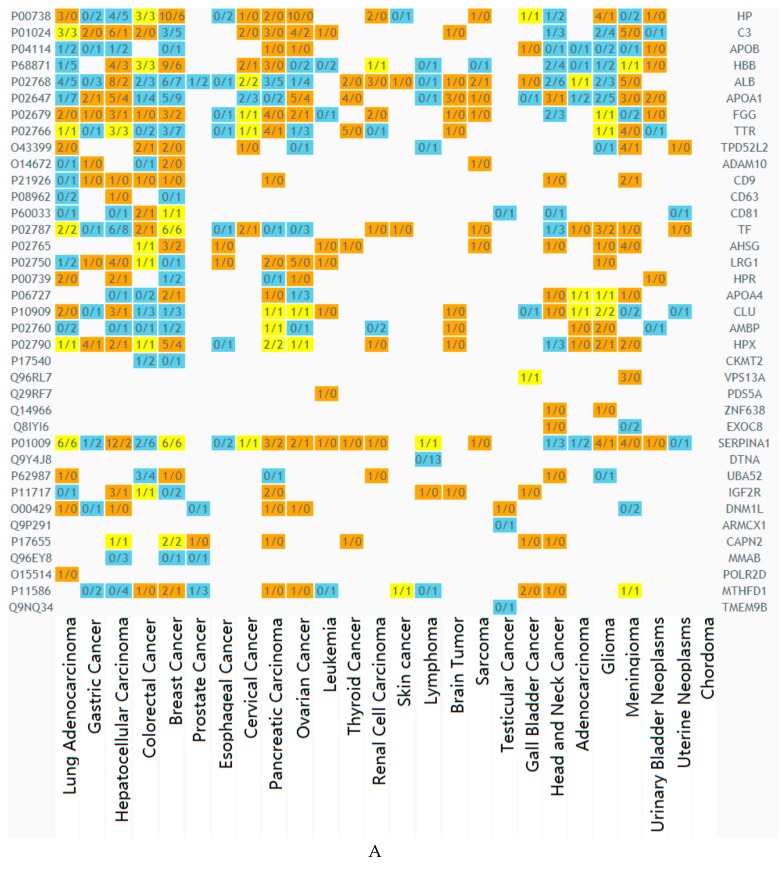

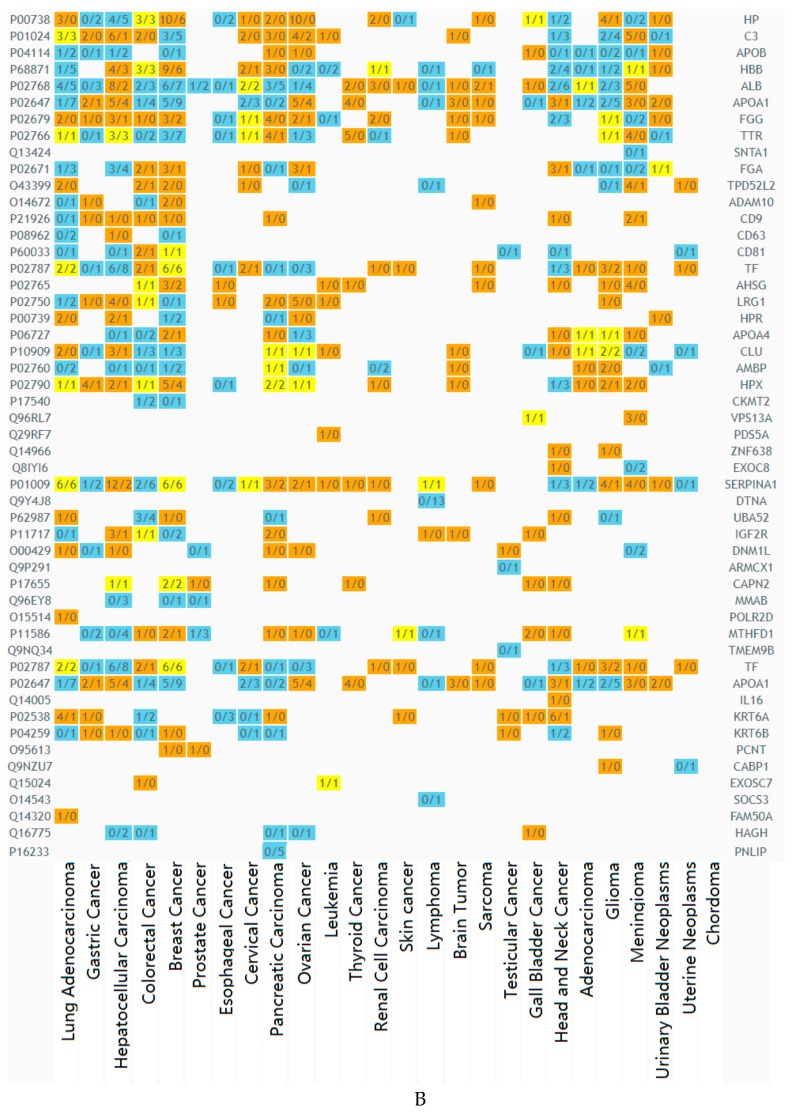
Heat map generated by dbDEPC 3.0 [17] from exosomal proteins from BCP blood, associated with different cancer types; plasma exosomes (**A**), total blood exosomes (**B**). Orange means that the number of the studies identified this protein as up-regulated is more than the number of the studies identified the protein as down-regulated; blue means that the number of the studies identified this protein as up-regulated is less than the number of the studies identified protein as down-regulated; yellow means that the number of the studies identified the protein as up-regulated is equal to the number of the studies identified protein as down-regulated.

Using the dbDEPC 3.0 database (database of Differently Expressed Proteins in Human Cancer) [17] it was found that in plasma exosomes from BCPs, 49 (66%) proteins were associated with malignant neoplasms of which 38 (51%) were associated with the development of breast cancer (Figure 4A). It should be noted that in total blood exosomes from BCPs 86 (59%) proteins associated with malignant neoplasms was revealed of which 51 (35%) were associated with the development of breast cancer (Figure 4B). Thus, breast cancer-associated proteins in plasma exosomes were detected more frequently than in total blood exosomes (51% vs. 35%), what can be explained by the intensive study of the composition of plasma exosomes and the recently discovered phenomenon of exosomes associated with blood cells.

Hyper- and hypo-expressed proteins associated with breast cancer development were equally represented in the composition of total blood exosomes (39% and 43% hyper- and hypo-expressed proteins, respectively), however, hypo-expressed proteins (47% vs. 29% hyper-expressed proteins) predominate in plasma exosomes (Figure 4).

**Figure 5:**
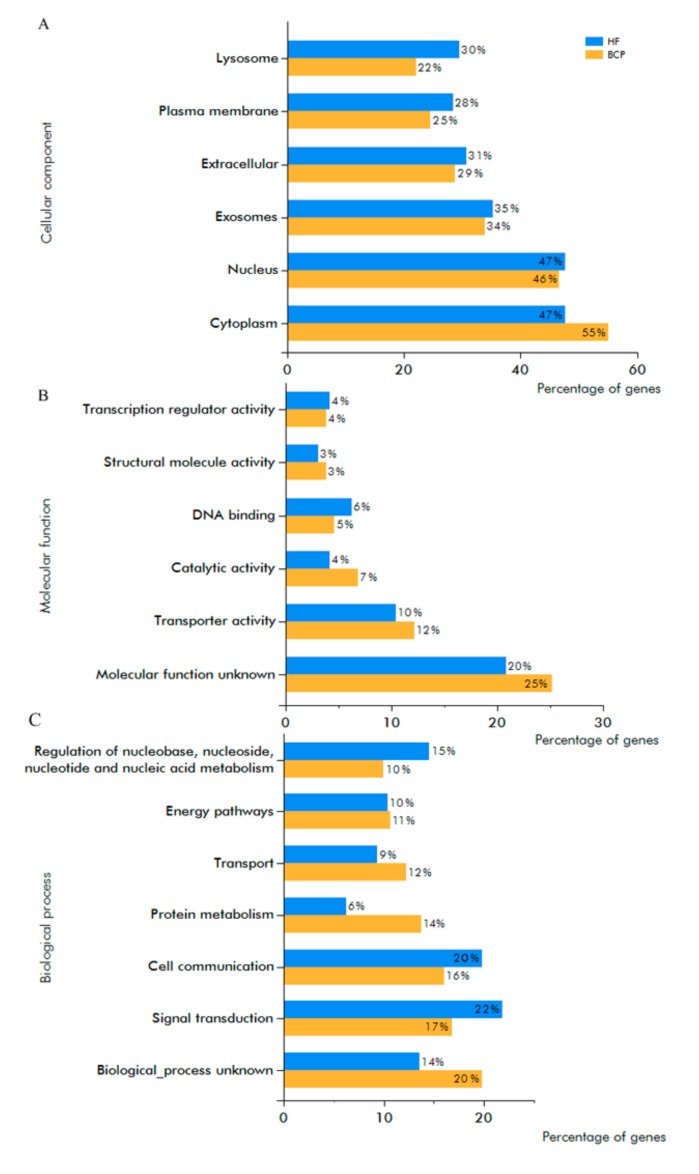
GO analysis of exosomal proteins from HF and BCP blood. Proteins were classified by cellular component (**A**), molecular function (**B**) and biological processes (**C**).

Functional enrichment analysis was performed using FunRich software for all exosomal proteins, revealing enriched GO terms for cellular components, molecular functions and biological processes (Figure 5). It was revealed that in exosomes from BCP blood, the share of cytoplasmic proteins increased in comparison to exosomes from HF blood (55% vs. 47%) (Figure 5A). The functions of 20% of exosomal proteins from HF blood and 25% of exosomal proteins from BCP blood were not clear; however, with the development of pathology, the portion of proteins with catalytic activity and transporter activity slightly increased (Figure 5B). Common exosomal proteins from HF and BCP blood exosomes were mainly involved in cell communication (20% and 16%, respectively), signal transduction (22% and 17%, respectively), and regulation of nucleoside, nucleotide, and nucleic acids metabolism (15% and 10%, respectively). It should also be noted that proteins involved in protein metabolism and transport proteins were found in exosomes from BCP blood more frequently (Figure 5C).

**Figure 6:**
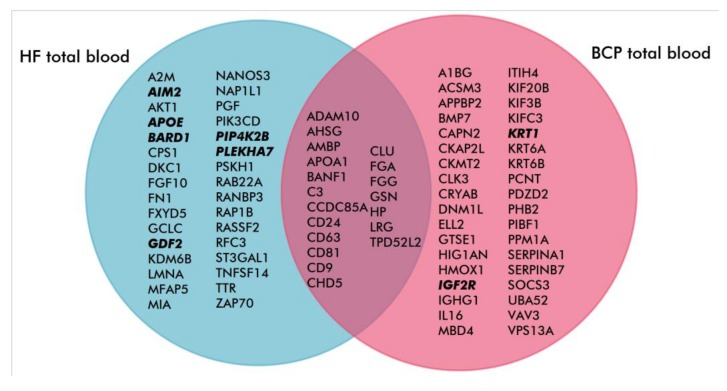
Venn–Euler diagram of exosomal proteins associated with invasion (blood from HFs and BCPs). Proteins that suppress invasion are in bold.

**Figure 7:**
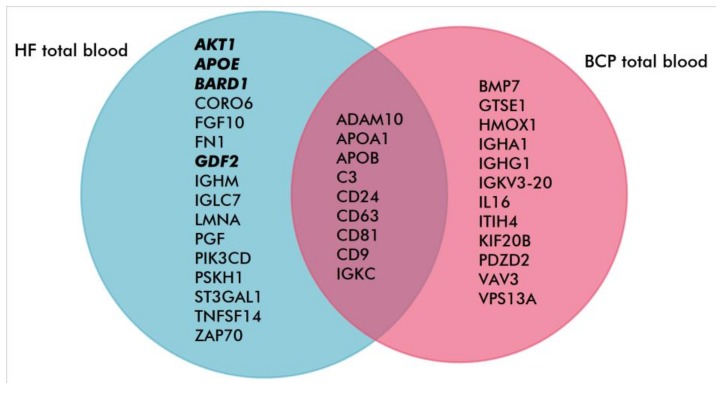
Venn–Euler diagram of exosomal proteins associated with cell migration (blood from HFs and BCPs). Proteins that suppress migration are in bold.

A literature analysis of exosomal proteins revealed that 51 (46%) were associated with invasion-associated functions in total blood exosomes from HFs, and 55 (38%) such proteins were detected in total BCP blood exosomes, 19 (22%) of which were common to both groups (Figure 6). Notably, in the proteome of HF exosomes, several proteins were engaged with invasion suppression (AIM2, APOE, BARD1, GDF2, PIP4K2B and PLEKHA7) while only two such proteins (IGF2R and KRT1) were revealed in exosomes from BCP blood. Furthermore, 25 (23%) and 21 (14%) proteins involved in the regulation of cell migration were identified in HF and BCP total blood exosomes, respectively (Figure 7). GO analysis using QuickGO revealed that four (APOE, AKT1, GDF2 and BARD1) out of 16 unique proteins from exosomes from HF blood were involved in the negative migration regulation of endothelial and lymphocytic cells [25,26,27]. Thus, it appeared that proteomic profiles of exosomes from HF blood were enriched by proteins for the negative regulation of cell migration and invasion.

### 3.5. ADAM-10 Expression Levels are Increased in Exosomes from BCP Luminal Subtype Blood

ADAM-10 is the most common exosomal metalloproteinase involved in the ectodomain shedding of various substrates, including growth factor receptors, adhesion receptors and cadherins [28]. This leads to increased cell motility and, as a consequence, an increase in the metastatic potential of tumor cells [29].

Since CD9 and CD24 were the most represented receptors on the surface of plasma and total blood exosomes, levels of the tetraspanin-associated metalloproteinase, ADAM-10 were estimated for CD9 and CD24-positive exosomes. The expression of ADAM-10 was significantly increased in subpopulations of CD9-positive exosomes, from plasma and total blood of luminal subtype BCPs, when compared with HF (the difference was 1.8 and 1.3 fold, respectively), and triple-negative subtype BCP samples (the difference was 1.6 and 1.2 fold, respectively). Moreover, in this subpopulation from total blood, ADAM-10 expression in BCPs with a luminal subtype was significantly reduced (1.5 times), when compared with plasma exosomes (Table 8). At the same time, analysis of ADAM-10 expression in CD24-positive exosomes from plasma and total blood did not reveal a significant difference of metalloproteinase levels between HF and BCP (Table 8).

## 4. Discussion

Interactions between cancer cells and their microenvironments are important for tumor development [8]. Communications between tumor and non-malignant cells can be performed by exosomes, which transfer molecules such as mRNAs, microRNAs and proteins between cells [8,30]. It should be noted that the majority of studies were performed with exosomes from cell culture. However, the pool of exosomes that circulate in the blood (blood circulating exosomes) is comprised of exosomes secreted by leukocytes, erythrocytes, thrombocytes, and endotheliocytes (i.e., either by hemic cells or cells interacting with the blood), as well as tissue cells, such as fibroblasts, epithelial cells and tumor cells [31,32]. Similarly noteworthy is the two fractions of exosomes in the blood: cell-free plasma exosomes and blood cell-surface-associated exosomes [11]. Undoubtedly, exosome transport is provided not only by the liquid blood fraction but by blood cells as well. However, the interactions of normal and metastatic tumor cells with cell-free exosomes and blood cell-associated exosomes have not yet been studied.

Here, we demonstrated that plasma exosomes morphologically resemble total blood exosomes and that both pools of exosomes are positive for CD24, CD63, and CD9 markers. However, the proportion of blood cell-associated exosomes in breast cancer is decreased, in comparison with the healthy state. Since most exosomes in the blood of cancer patients are of non-tumor origins, the reason for this decrease remains unclear. This phenomenon may be associated with an increased affinity of exosomes for blood cells, or reflect true decreases in exosome levels on the blood cell surface membrane.

One of the reasons for decreases in exosome levels on the surface of BCP blood cells may be the increased activity of sheddases directly interacting not only with extracellular matrix proteins but with membrane receptors and cell adhesion molecules to target cells [33].

Since exosomal surfaces contain CD9 and CD24 receptors, the level of tetraspanin-associated metalloproteinase ADAM-10 was estimated for CD9 and CD24-positive exosomes. ADAM-10 levels were significantly increased in subpopulations of CD9-positive exosomes isolated from the plasma and total blood fractions of patients with luminal breast cancer, as compared with HFs and triple-negative subtype BCPs. At the same time, there were no significant changes in ADAM-10 expression levels on CD24-positive exosomes. These results correlate with data [34] suggesting that the inclusion of the mature form of ADAM-10 into exosomes, and the proteolytic activity of this sheddase is regulated by the tetraspanins, CD9, CD81 and CD82. Thus, our data suggest that increased ADAM-10 expression levels on the surface of exosomes reduce their binding to blood cells [28], thereby increasing concentrations of cell-free exosomes, which in turn allows exosomes to act as intermediaries in providing intercellular communication through horizontal transfer of RNA and proteins, affecting the development of both primary neoplasms and distant metastases.

In addition to the effects of cancer exosomes on healthy tissue cells, the effects of exosomes secreted by normal tissue cells on tumor cells are also of importance in disease pathogenesis. For example, cells from a growing tumor are affected by exosomes secreted by normal cells in the surrounding stroma, and circulating tumor cells are affected by plasma exosomes of different origins. Therefore, we sought to examine the effects of plasma exosomes and total blood exosomes from HF and BCP blood on cell migration, a key step in metastasis. We found that exosomes from BCP total blood significantly stimulate non-tumor cell migration. This effect manifests itself as an increased number of motile cells; however, we did not observe any influence of exosomes on migratory cell velocity. Exosomes from plasma and total blood, obtained from both HFs and BCPs, stimulated the migration velocity of tumor cell but did not affect the amount of the migrating cells. Total blood exosomes from HFs had the least effect on the migration velocity.

To unravel molecular regulatory roles behind blood exosome-mediated migration and invasion, protein profiling on plasma and total blood exosomes was performed. Currently, there are few reports on the proteomic analysis of exosomes, especially for plasma exosomes in breast cancer, when compared to other types of cancer [35,36] and a dearth of data on proteomic profiles in blood cell-bound exosomes. Technical difficulties in blood exosome proteomics are primarily associated with the heterogeneity of the composition of circulating vesicles. 

Consistent with previous studies [5,37] our analysis showed that the majority of proteins from cell-free and total blood exosomes were of cytoplasmic origins. An increase in proteins involved in protein metabolism, and proteins with transport and catalytic activities in the blood exosomes of BCPs, suggest a significant role of exosomes in the development of malignant neoplasms. We did not find protein inhibitors of cell migration in these exosomes that correlated with the phenomena of stimulation of non-tumor MCF10A cells migrations. At the same time, the presence of cell migration inhibitors (APOE, AKT1, BARD1, GDF2) in exosomes from blood HFs accompanied by low stimulating effects on the migration velocity of tumor cells. Thus, the analysis of protein composition in exosomes complements their biogenesis and functional roles in cancer development.

## 5. Conclusions 

In conclusion, this study indicates that the increased concentration of cell-free exosomes in the blood from BCPs correlated with increased exosomal ADAM-10 expression; however, total blood exosomes contain more malignant neoplasm-associated proteins than plasma exosomes. Exosomes from the total blood of BCPs were enriched by proteins with catalytic, transporter and protein metabolism activities. In this first proteomic profiling study of exosomes from plasma and total blood from HFs and BCPs, exosomes from HF total blood were enriched for proteins inhibiting cell migration and invasion, with low stimulating activity on the migration velocity of cancer cells. Follow-up studies are required to understand protein inhibitors of cell migration (i.e., APOE, AKT1, BARD1 and GDF2) and their usefulness in developing new anti-metastatic therapies.

## Figures and Tables

**Table 1:** General Clinical Characteristics of Breast Cancer Patients.

		No (%)
**Tumor Stage**	T1	11 (48)
	T2	9 (39)
	T3	1 (4)
	T4	2 (9)
Nodal Status	N0	14 (61)
	N1	7 (30)
	N2	1 (4)
	N3	1 (4)
	M0	23 (100)
Receptor Status	ER and/or Pr +	15 (65)
	ER and Pr -	5 (22)
	Unknown	3 (13)
HER2 Status	Positive	15 (65)
	Negative	5 (22)
	Unknown	3 (13)
Ki-67	< 10	4 (17)
	10 ≤ ≤ 20	6 (26)
	> 20	10 (43)
	Unknown	3 (13)
Histologic Grade	II	16 (70)
	III	2 (9)
	Unknown	5 (22)
Infiltrative Ductal Carcinoma		23 (100%)
Total Patients		23 (100%)

**Table 2:** Concentration and Size-distribution of Plasma Exosomes and Total Blood Exosomes Isolated from the Blood of HFs and BCPs. Data of NTA (Malvern, NS-300).

1. Source of Exosomes		Median and Range of Exosomes Concentration × 10^7^/ml of blood	Median ± SE of Exosome Size, nm
HFs	Plasma	8 4–10	96 ± 16
	Total Blood	26 25–27	130 ± 5
BCPs Luminal Subtype	Plasma	23 5–88	127 ± 7
	Total Blood	24 7–92	129 ± 12
BCPs Triple-negative Subtype	Plasma	19 9–30	103 ± 72
	Total Blood	31 28–63	131 ± 95

**Table 3:** Expression of CD 24, CD 63 and CD 81 on the Surface of Exosomes from Plasma and Total Blood of HFs and BCPs*

2. Source of Exosomes		CD9-Positive Exosomes		CD24-Positive Exosomes	
		CD 63 Expression	CD 81 Expression	CD 9 Expression	CD 63 Expression
HFs	Plasma	513 ± 76	645 ± 97	1048 ± 120	523 ± 75
	Total blood	523 ± 42	698 ± 63	832 ± 81	540 ± 44
BCPs Luminal Subtype	Plasma	464 ± 48	630 ± 67	1078 ± 116	470 ± 56
	Total blood	493 ± 47	689 ± 70	1153 ± 110	494 ± 48
BCPs Triple-negative Subtype	Plasma	515 ± 52	688 ± 48	859 ± 85	550 ± 53
	Total blood	543 ± 43	704 ± 49	742 ± 67	527 ± 58

*, data represent median fluorescence intensity (MFI) ± SEM.

**Table 4:** The Proportion of Motile Cells in Response to Exosome Treatment.

Source of Exosomes	Percentage of Migrating Cells, Median and 25–75% Percentile	
	MCF10A Cells	SKBR-3 Cells
Negative Control	6.5 3.0–7.3 –	2.0 0.5–3 –
Positive Control	96.5 95.8–100 *p* < 0.0001	15.0 14.0–16.0 *p* = 0.0024
from HF Plasma	9.0 7.3–11.5 *p* = 0.0399	6.5 3.3–9.8 NS
from HF Total Blood	12.0 4.3–13.5 NS	5.0 1.5–6.5 NS
from BCP Plasma	17.0 4.3–17.8 *p* = 0.0179	6.0 4.5–8.0 NS
from BCP Total Blood	24.0 13.8–26.3 *p* = 0.0097	5.0 1.0–6.0 NS

**Table 5:** Universal Exosomal Proteins Identified in the Blood of HFs and BCPs*.

Uniprot ID	Protein Name	Gene Name	ExoCarta	Score
**O14672**	Disintegrin and metalloproteinase domain-containing protein 10	ADAM10	+	60
**O43399**	Tumor protein D54	TPD52L2	+	61
**O75531**	Barrier-to-autointegration factor	BANF1	+	57
**P00738**	Haptoglobin	HP	+	74
**P00739**	Haptoglobin-related protein	HPR	−	60
**P01024**	Complement C3	C3	+	137
**P01834**	Ig kappa chain C region	IGKC	+	59
**P01859**	Ig gamma-2 chain C region	IGHG2	+	59
**P02647**	Apolipoprotein A-I	APOA1	+	176
**P02671**	Fibrinogen alpha chain	FGA	+	57
**P02675**	Fibrinogen beta chain	FGB	+	60
**P02679**	Fibrinogen gamma chain	FGG	+	67
**P02750**	Leucine-rich alpha-2-glycoprotein	LRG	−	60
**P02760**	Alpha-1-microglycoprotein	AMBP	+	60
**P02765**	Alpha-2-HS-glycoprotein	AHSG	+	60
**P02766**	Transthyretin	TTR	+	58
**P02768**	Serum albumin	ALB	+	149
**P02787**	Serotransferrin	TF	−	137
**P02790**	Hemopexin	HPX	+	60
**P04114**	Apolipoprotein B-100	APOB	+	56
**P06396**	**Gelsolin**	GSN	−	60
**P06727**	Apolipoprotein A-IV	APOA4	+	60
**P08962**	CD63 antigen	CD63	+	60
**P10909**	Clusterin	CLU	+	60
**P21926**	CD9 antigen	CD9	+	60
**P25063**	Signal transducer CD24	CD24	+	60
**P60033**	CD81 antigen	CD81	+	60
**P68871**	Hemoglobin subunit beta	HBB	+	72
**Q08426**	**Peroxisomalbifunctional enzyme**	EHHADH	+	56
**Q13424**	Alpha-1-syntrophin	SNTA1	+	60
**Q15776**	Zinc finger protein with KRAB and SCAN domains 8	ZKSCAN8	−	56
**Q8TES7**	Fas-binding factor 1	FBF1	+	56
**Q96PX6**	Coiled-coil domain-containing protein 85A	CCDC85A	−	64
**Q9H6Z4**	**Ran-binding protein 3**	RANBP3	+	56

* - proteins unique to the total blood fraction are in bold.

**Table 6:** Unique Exosomal Proteins Identified in Total Blood of HFs.

Uniprot ID	Protein Name	Gene Name	ExoCarta	Score
**A6NCL7**	Ankyrin repeat domain-containing protein 33B	ANKRD33B	−	57
**A8MU93**	Uncharacterizedprotein C17orf100	C17orf100	−	56
**O15054**	Lysine-specific demethylase 6B	KDM6B	+	58
**O60832**	H/ACA ribonucleoprotein complex subunit 4	DKC1	+	56
**O95602**	DNA-directed RNA polymerase I subunit RPA1	POLR1A	−	57
**P02545**	Prelamin-A/C	LMNA	+	59
**P05976**	Myosin light chain 1/3, skeletal muscle isoform	MYL1	+	56
**P26440**	Isovaleryl-CoA dehydrogenase, mitochondrial	IVD	−	57
**P31327**	Carbamoyl-phosphate synthase [ammonia], mitochondrial	CPS1	+	62
**P31749**	RAC-alpha serine/threonine-protein kinase	AKT1	+	62
**P48506**	Glutamate-cysteine ligase catalytic subunit	GCLC	−	56
**P49748**	Very long-chain specific acyl-CoA dehydrogenase, mitochondrial	ACADVL	−	56
**P49763**	Placenta growth factor	PGF	−	56
**P53674**	Beta-crystallin B1	CRYBB1	−	80
**Q11201**	CMP-N-acetylneuraminate-beta-galactosamide-alpha-2,3-sialyltransferase 1	ST3GAL1	−	58
**Q49A33**	Putative zinc finger protein 876	ZNF876P	−	56
**Q49MG5**	Microtubule-associated protein 9	MAP9	−	58
**Q504T8**	Midnolin	MIDN	−	56
**Q69YQ0**	Cytospin-A	SPECC1L	+	68
**Q6P1J9**	Parafibromin	CDC73	−	60
**Q6ZS02**	Putative GED domain-containing protein DNM1P46	DNM1P46	−	57
**Q7Z553**	MAM domain-containing glycosylphosphatidylinositol anchor protein 2	MDGA2	−	56
**Q86VE0**	Myb-related transcription factor, partner of profilin	MYPOP	−	57
**Q8N8C0**	Zinc finger protein 781	ZNF781	−	65
**Q9HBI5**	Uncharacterized protein C3orf14	C3orf14	−	56
**Q9P2W7**	Galactosylgalactosylxylosylprotein 3-beta-glucuronosyltransferase 1	B3GAT1	−	56
**Q9UK05**	Growth/differentiation factor 2	GDF2	+	59

**Table 7:** Unique Exosomal Proteins Identified in Total Blood of BCPs.

UniprotID	Protein Name	Gene Name	Exocarta	Score
**A0A0B4J1X5**	Immunoglobulin heavy variable 3-74	IGHV3-74	−	56
**A0A1B0GVM6**	Uncharacterized protein C11orf97	C11orf97	−	56
**A0A589**	T cell receptor beta variable	TRBV4-3	−	56
**O14543**	Suppressor of cytokine signaling 3	SOCS3	−	63
**O15018**	PDZ domain-containing protein 2	PDZD2	−	57
**O15020**	Spectrin beta chain, non-erythrocytic 2	SPTBN2	+	66
**O15083**	ERC protein 2	ERC2	−	56
**O60397**	Putative cytochrome c oxidase subunit 7A3, mitochondrial	COX7A2P2	−	58
**O75635**	Serpin B7	SERPINB7	−	56
**O95243**	Methyl-CpG-binding domain protein 4	MBD4	−	56
**O95613**	Pericentrin	PCNT	−	71
**P02538**	Keratin, type II cytoskeletal 6A	KRT6A	+	62
**P04217**	Alpha-1B-glycoprotein	A1BG	+	128
**P04259**	Keratin, type II cytoskeletal 6B	KRT6B	+	58
**P04264**	Keratin, type II cytoskeletal 1	KRT1	−	81
**P09601**	Heme oxygenase 1	HMOX1	−	56
**P13497**	Bone morphogenetic protein 1	BMP1	−	60
**P16233**	Pancreatic triacylglycerol lipase	PNLIP	−	56
**P35527**	Keratin, type I cytoskeletal 9	KRT9	+	57
**P48167**	Glycine receptor subunit beta	GLRB	−	56
**P49761**	Dual specificity protein kinase CLK3	CLK3	−	56
**P50440**	Glycine amidinotransferase, mitochondrial	GATM	−	56
**P55199**	RNA polymerase II elongation factor ELL	ELL	−	56
**P62987**	Ubiquitin-60S ribosomal protein L40	UBA52	−	57
**Q13522**	Protein phosphatase 1A	PPM1A	+	56
**Q13535**	Serine/threonine-protein kinase ATR	ATR	−	58
**Q14005**	Pro-interleukin-16	IL16	−	56
**Q14320**	Protein FAM50A	FAM50A	−	61
**Q14571**	Inositol 1,4,5-trisphosphate receptor type 2	ITPR2	+	67
**Q14624**	Inter-alpha-trypsin inhibitor heavy chain H4	ITIH4	+	80
**Q15024**	Exosome complex component RRP42	EXOSC7	+	56
**Q15477**	Helicase SKI2W	SKIV2L	+	65
**Q16775**	Hydroxyacylglutathione hydrolase, mitochondrial	HAGH	+	56
**Q16890**	Tumor protein D53	TPD52L1	−	56
**Q2M218**	Zinc finger protein 630	ZNF630	−	80
**Q4G0S7**	Coiled-coil domain-containing protein 152	CCDC152	−	63
**Q52M93**	Zinc finger protein 585B	ZNF585B	−	57
**Q5R372**	Rab GTPase-activating protein 1-like	RABGAP1L	−	56
**Q5VWM5**	PRAME family member 9/15	PRAMEF9	−	58
**Q68J44**	Dual specificity phosphatase DUPD1	DUPD1	−	57
**Q6P1J6**	Phospholipase B1, membrane-associated	PLB1	−	56
**Q7L5N7**	Lysophosphatidylcholine acyltransferase 2	LPCAT2	−	56
**Q7L5Y9**	E3 ubiquitin-protein transferase MAEA	MAEA	−	56
**Q7RTT3**	Putative protein SSX9	SSX9P	−	189
**Q8IUS5**	Epoxide hydrolase 4	EPHX4	−	57
**Q8IYA6**	Cytoskeleton-associated protein 2-like	CKAP2L	−	65
**Q8IYE0**	Coiled-coil domain-containing protein 146	CCDC146	−	57
**Q8NDD1**	Uncharacterizedprotein C1orf131	C1orf131	−	56
**Q8NEQ6**	Steroid receptor-associated and regulated protein	SRARP	−	59
**Q8WXR4**	Myosin-IIIb	MYO3B	+	56
**Q8WXS5**	Voltage-dependent calcium channel gamma-8 subunit	CACNG8	−	63
**Q92622**	Run domain Beclin-1-interacting and cysteine-rich domain-containing protein	RUBCN	−	56
**Q92624**	Amyloid protein-binding protein 2	APPBP2	−	61
**Q99623**	Prohibitin-2	PHB2	+	74
**Q9H497**	Torsin-3A	TOR3A	+	63
**Q9H4Q4**	PR domain zinc finger protein 12	PRDM12	−	56
**Q9H9E3**	Conserved oligomeric Golgi complex subunit 4	COG4	−	56
**Q9NQG6**	Mitochondrial dynamics protein MID51	MIEF1	−	60
**Q9NSD9**	Phenylalanine-tRNA ligase beta subunit	FARSB	+	56
**Q9NZJ4**	Sacsin	SACS	−	56
**Q9NZU7**	Calcium-binding protein 1	CABP1	−	56
**Q9Y4E5**	E3 SUMO-protein ligase ZNF451	ZNF451	+	69

**Table 8:** Expression of CD9 and CD24 on the Surface of ADAM-10 Positive Exosomes from Plasma and Total Blood of HFs and BCPs.

4. Source of Exosomes		CD9+	CD24+
HFs	Plasma	844 ± 99	884 ± 124
	Total blood	804 ± 79	920 ± 91
BCPs Luminal subtype	Plasma	1531 ± 162*	926 ± 94
	Total blood	1026 ± 100*	1103 ± 99
BCPs Triple-negative subtype	Plasma	962 ± 88	908 ± 109
	Total blood	862 ± 69	969 ± 106

*, differences were significant compared to HFs (*p* value < 0.05). Data represent median fluorescence intensity (MFI) ± SEM.

## Supplementary Materials

The following are available online at https://www.mdpi.com/2218-273X/10/4/495/s1, Figure S1: title, Figure S2: title, Table S1: title, Table S2: title
