# Supplementary material for: Proteomic Analysis of Blood Exosomes from Healthy Females and Breast Cancer Patients Reveals an Association between Different Exosomal Bioactivity on Non-tumorigenic Epithelial Cell and Breast Cancer Cell Migration in Vitro
Source: Biomolecules. 2020 Mar 25;10(4):495. doi: 10.3390/biom10040495 (PMC7226042; doi:10.3390/biom10040495)
Supplement: Supplementary file 1 [file biomolecules-10-00495-s001.zip › suppl files/Legend to Suppl Fig 1.docx]

Suppl. Fig. 1. Trajectory plots of single-cell migration experiments of MCF10A (A) and SKBR-3 (B) in presence or absence of exosomes.
