# Supplementary material for: Proteomic Analysis of Blood Exosomes from Healthy Females and Breast Cancer Patients Reveals an Association between Different Exosomal Bioactivity on Non-tumorigenic Epithelial Cell and Breast Cancer Cell Migration in Vitro
Source: Biomolecules. 2020 Mar 25;10(4):495. doi: 10.3390/biom10040495 (PMC7226042; doi:10.3390/biom10040495)
Supplement: Supplementary file 1 [file biomolecules-10-00495-s001.zip › suppl files/Legend to Suppl Fig 2.docx]

Suppl. Fig. 2. Analysis of exosomal proteins by gradient Laemmli 10–20% electrophoresis. The gel was stained with Coomassie R250: 1, molecular weight protein ladder (Thermo Scientific PageRuler Plus Prestained Protein Ladder); 2, exosomes from plasma of HFs; 3, exosomes from plasma of BCPs; 4, exosomes from total blood of HFs; 5, exosomes from total blood of BCPs.
