# Supplementary material for: Proteomic Analysis of Blood Exosomes from Healthy Females and Breast Cancer Patients Reveals an Association between Different Exosomal Bioactivity on Non-tumorigenic Epithelial Cell and Breast Cancer Cell Migration in Vitro
Source: Biomolecules. 2020 Mar 25;10(4):495. doi: 10.3390/biom10040495 (PMC7226042; doi:10.3390/biom10040495)
Supplement: Supplementary file 1 [file biomolecules-10-00495-s001.zip › suppl files/Suppl Table 1-rev.docx]

Suppl. Table 1.Exosomal proteins identified in plasma and total blood of HFs*

| UniprotID | Protein name | Gene Name | Exo Carta | Score |
| --- | --- | --- | --- | --- |
| A0A1B0GU71 | Uncharacterized protein CFAP97D2 | CFAP97D2 | - | 56 |
| A0M8Q6 | Ig lambda-7 chain C region | IGLC7 | + | 67 |
| **A6NCL7** | **Ankyrin repeat domain-containing protein 33B** | ANKRD33B | - | 57 |
| **A8MU93** | **Uncharacterized protein C17orf100** | C17orf100 | - | 56 |
| F8VUA8 | Uncharacterized protein CLBA1 | CLBA1 | - | 72 |
| H0YGS3 | Microfibrillar-associated protein 5 | MFAP5 | + | 56 |
| O00329 | Phosphatidylinositol 4,5-bisphosphate 3-kinase catalytic subunit delta isoform | PIK3CD | - | 58 |
| O14672 | *Disintegrin and metalloproteinase domain-containing protein 10* | ADAM10 | + | 60 |
| O14862 | Interferon-inducible protein AIM2 | AIM2 | - | 57 |
| **O15054** | **Lysine-specific demethylase 6B** | KDM6B | + | 58 |
| O15520 | Fibroblast growth factor 10 | FGF10 | - | 61 |
| O43309 | Zinc finger and SCAN domain-containing protein 12 | ZSCAN12 | - | 56 |
| O43399 | *Tumor protein D54* | TPD52L2 | + | 61 |
| O43557 | Tumor necrosis factor ligand superfamily member 14 | TNFSF14 | - | 57 |
| O43678 | NADH dehydrogenase [ubiquinone] 1 alpha subcomplex subunit 2 | NDUFA2 | - | 56 |
| **O60832** | **H/ACA ribonucleoprotein complex subunit 4** | DKC1 | + | 56 |
| O60861 | Growth arrest-specific protein 7 | GAS7 | + | 56 |
| O75526 | RNA-binding motif protein, X-linked-like-2 | RBMXL2 | - | 56 |
| O75531 | *Barrier-to-autointegration factor* | BANF1 | + | 57 |
| **O95602** | **DNA-directed RNA polymerase I subunit RPA1** | POLR1A | - | 57 |
| O95831 | Apoptosis-inducing factor 1, mitochondrial | AIFM1 | - | 59 |
| O96004 | Heart- and neural crest derivatives-expressed protein 1 | HAND1 | - | 56 |
| P00738 | *Haptoglobin* | HP | + | 74 |
| P00739 | *Haptoglobin-related protein* | HPR | + | 60 |
| P01023 | Alpha-2-macroglobulin | A2M | + | 178 |
| P01024 | *Complement C3* | C3 | + | 137 |
| P01834 | *Ig kappa chain C region* | IGKC | + | 59 |
| P01859 | *Ig gamma-2 chain C region* | IGHG2 | + | 59 |
| P01871 | Ig mu chain C region | IGHM | + | 79 |
| **P02545** | **Prelamin-A/C** | LMNA | + | 59 |
| P02647 | *Apolipoprotein A-I* | APOA1 | + | 176 |
| P02649 | Apolipoprotein E | APOE | + | 60 |
| P02671 | *Fibrinogen alpha chain* | FGA | + | 57 |
| P02675 | *Fibrinogen beta chain* | FGB | + | 60 |
| P02679 | *Fibrinogen gamma chain* | FGG | + | 67 |
| P02750 | *Leucine-rich alpha-2-glycoprotein* | LRG | + | 60 |
| P02751 | Fibronectin | FN | + | 115 |
| P02760 | *Alpha-1-microglycoprotein* | AMBP | + | 60 |
| P02765 | *Alpha-2-HS-glycoprotein* | AHSG | + | 60 |
| P02766 | *Transthyretin* | TTR | + | 58 |
| P02768 | *Serum albumin* | ALB | + | 149 |
| **P02787** | ***Serotransferrin*** | **TF** | **-** | **137** |
| P02790 | *Hemopexin* | HPX | + | 60 |
| P04114 | Apolipoprotein B-100 | APOB | + | 68 |
| **P05976** | **Myosin light chain 1/3, skeletal muscle isoform** | MYL1 | + | 56 |
| P06396 | *Gelsolin* | GSN | + | 60 |
| P06727 | *Apolipoprotein A-IV* | APOA4 | + | 60 |
| P08603 | Complement factor H | CFH | + | 85 |
| P08962 | *CD63 antigen* | CD63 | + | 60 |
| P10909 | *Clusterin* | CLU | + | 60 |
| P11801 | Serine/threonine-protein kinase H1 | PSKH1 | - | 98 |
| P12074 | Cytochrome c oxidase subunit 6A1, mitochondrial | COX6A1 | - | 56 |
| P21926 | *CD9 antigen* | CD9 | + | 60 |
| P23109 | AMP deaminase 1 | AMPD1 | - | 59 |
| P25063 | *Signal transducer CD24* | CD24 | + | 60 |
| **P26440** | **Isovaleryl-CoA dehydrogenase, mitochondrial** | IVD | - | 57 |
| **P31327** | **Carbamoyl-phosphate synthase [ammonia], mitochondrial** | CPS1 | + | 62 |
| **P31749** | **RAC-alpha serine/threonine-protein kinase** | AKT1 | + | 62 |
| P40938 | Replication factor C subunit 3 | RFC3 | + | 64 |
| P43403 | Tyrosine-protein kinase ZAP-70 | ZAP70 | + | 64 |
| **P48506** | **Glutamate--cysteine ligase catalytic subunit** | GCLC | - | 56 |
| **P49748** | **Very long-chain specific acyl-CoA dehydrogenase, mitochondrial** | ACADVL | - | 56 |
| **P49763** | **Placenta growth factor** | PGF | - | 56 |
| P50749 | Ras association domain-containing protein 2 | RASSF2 | + | 56 |
| **P53674** | **Beta-crystallin B1** | CRYBB1 | - | 80 |
| P55209 | Nucleosome assembly protein 1-like 1 | NAP1L1 | + | 56 |
| P56373 | P2X purinoceptor 3 | P2RX3 | - | 60 |
| P60033 | *CD81 antigen* | CD81 | + | 60 |
| P60323 | Nanos homolog 3 | NANOS3 | - | 56 |
| P61201 | COP9 signalosome complex subunit 2 | COPS2 | + | 57 |
| P61224 | Ras-related GTP-binding protein B | RAP1B | + | 56 |
| P68871 | *Hemoglobin subunit beta* | HBB | + | 72 |
| P78356 | Phosphatidylinositol 4-kinase type 2-beta | PIP4K2B | + | 60 |
| **Q08426** | ***Peroxisomal bifunctional enzyme*** | EHHADH | + | 56 |
| **Q11201** | **CMP-N-acetylneuraminate-beta-galactosamide-alpha-2,3-sialyltransferase 1** | ST3GAL1 | + | 58 |
| Q13424 | *Alpha-1-syntrophin* | SNTA1 | + | 60 |
| Q15776 | *Zinc finger protein with KRAB and SCAN domains 8* | ZKSCAN8 ZNF192 | - | 56 |
| Q16674 | Melanoma-derived growth regulatory protein | MIA | - | 56 |
| **Q49A33** | **Putative zinc finger protein 876** | ZNF876P | - | 56 |
| **Q49MG5** | **Microtubule-associated protein 9** | MAP9 | - | 58 |
| Q4LEZ3 | Alanine and arginine-rich domain-containing protein | AARD | - | 57 |
| **Q504T8** | **Midnolin** | MIDN | - | 56 |
| **Q69YQ0** | **Cytospin-A** | SPECC1L | + | 68 |
| Q6IQ23 | Pleckstrin homology domain-containing family A member 7 | PLEKHA7 | + | 56 |
| **Q6P1J9** | **Parafibromin** | CDC73 | - | 60 |
| Q6QEF8 | Coronin-6 | CORO6 | - | 57 |
| **Q6ZS02** | **Putative GED domain-containing protein DNM1P46** | DNM1P46 | - | 57 |
| **Q7Z553** | **MAM domain-containing glycosylphosphatidylinositol anchor protein 2** | MDGA2 | - | 56 |
| **Q86VE0** | **Myb-related transcription factor, partner of profilin** | MYPOP | - | 57 |
| Q8N159 | N-acetylglutamate synthase, mitochondrial | NAGS | - | 66 |
| Q8N5S9 | Calcium/calmodulin-dependent protein kinase kinase 1 | CAMKK1 | - | 56 |
| **Q8N8C0** | **Zinc finger protein 781** | ZNF781 | - | 65 |
| Q8TDF6 | RAS guanyl-releasing protein 4 | RASGRP4 | - | 66 |
| Q8TDI0 | Chromodomain-helicase-DNA-binding protein 5 | CHD5 | - | 57 |
| Q8TES7 | *Fas-binding factor 1* | FBF1 | + | 56 |
| Q8WXH6 | Ras-related protein Rab-40A | RAB40A | - | 56 |
| Q969K3 | E3 ubiquitin-protein ligase RNF34 | ENF34 | - | 59 |
| Q96DB9 | FXYD domain-containing ion transport regulator 5 | FXYD5 | - | 56 |
| Q96PX6 | *Coiled-coil domain-containing protein 85A* | CCDC85A | - | 64 |
| Q99728 | BRCA1-associated RING domain protein 1 | BARD1 | - | 56 |
| Q9GZT8 | NIF3-like protein 1 | NIF3L1 | + | 56 |
| **Q9H6Z4** | ***Ran-binding protein 3*** | RANBP3 | + | 56 |
| **Q9HBI5** | **Uncharacterized protein C3orf14** | C3orf14 | - | 56 |
| Q9HCQ7 | Pro-FMRFamide-related neuropeptide VF | NPVF | - | 56 |
| Q9NUR3 | Transmembrane protein 74B | TMEM74B | - | 60 |
| **Q9P2W7** | **Galactosylgalactosylxylosylprotein 3-beta-glucuronosyltransferase 1** | **B3GAT1** | **-** | **56** |
| Q9UJW7 | Zinc finger protein 229 | ZNF229 | - | 58 |
| **Q9UK05** | **Growth/differentiation factor 2** | GDF2 | + | 59 |
| Q9UL26 | Ras-related protein Rab-22A | RAB22A | + | 64 |
| Q9Y2P0 | Zinc finger protein 835 | ZNF835 | - | 110 |

* - Universal proteins are in italics, proteins unique to the total blood fraction are in bold.
