# Supplementary material for: Proteomic Analysis of Blood Exosomes from Healthy Females and Breast Cancer Patients Reveals an Association between Different Exosomal Bioactivity on Non-tumorigenic Epithelial Cell and Breast Cancer Cell Migration in Vitro
Source: Biomolecules. 2020 Mar 25;10(4):495. doi: 10.3390/biom10040495 (PMC7226042; doi:10.3390/biom10040495)
Supplement: Supplementary file 1 [file biomolecules-10-00495-s001.zip › suppl files/Suppl Table 2-rev.docx]

Suppl. Table 2.Exosomal proteins identified in plasma and total blood of BCPs*

| UniprotID | Protein name | Gene Name | Exo Carta | Score |
| --- | --- | --- | --- | --- |
| **A0A0B4J1X5** | **Immunoglobulin heavy variable 3-74** | IGHV3-74 | - | 56 |
| A0A140G945 | Alpha-crystallin A2 chain | CRYAA2 | - | 56 |
| **A0A1B0GVM6** | **Uncharacterized protein C11orf97** | C11orf97 | - | 56 |
| **A0A589** | **T cell receptor beta variable** | TRBV4-3 | - | 56 |
| O00429 | Dynamin-1-like protein | DNM1L | + | 57 |
| O00555 | Voltage-dependent P/Q-type calcium channel subunit alpha-1A | CACNA1A | - | 60 |
| **O14543** | **Suppressor of cytokine signaling 3** | SOCS3 | - | 63 |
| O14672 | *Disintegrin and metalloproteinase domain-containing protein 10* | ADAM10 | + | 60 |
| **O15018** | **PDZ domain-containing protein 2** | PDZD2 | - | 57 |
| **O15020** | **Spectrin beta chain, non-erythrocytic 2** | SPTBN2 | + | 66 |
| O15050 | TPR and ankyrin repeat-containing protein 1 | TRANK1 | + | 65 |
| O15066 | Kinesin-like protein KIF3B | KIF3B | + | 56 |
| **O15083** | **ERC protein 2** | ERC2 | - | 56 |
| O15514 | DNA-directed RNA polymerase II subunit RPB4 | POLR2D | - | 56 |
| O43399 | *Tumor protein D54* | TPD52L2 | + | 61 |
| **O60397** | **Putative cytochrome c oxidase subunit 7A3, mitochondrial** | COX7A2P2 | - | 58 |
| O75027 | ATP-binding cassette sub-family B member 7, mitochondrial | ABCB7 | - | 59 |
| O75531 | *Barrier-to-autointegration factor* | BANF1 | + | 60 |
| **O75635** | **Serpin B7** | SERPINB7 | - | 56 |
| **O95243** | **Methyl-CpG-binding domain protein 4** | MBD4 | - | 56 |
| **O95613** | **Pericentrin** | PCNT | - | 71 |
| P00738 | *Haptoglobin* | HP | + | 135 |
| P00739 | *Haptoglobin-related protein* | HPR | + | 61 |
| P01009 | Alpha-1-antitrypsin | SERPINA1 | + | 192 |
| P01024 | *Complement C3* | C3 | + | 163 |
| P01619 | Immunoglobulin kappa light chain | IGKV3-20 | + | 58 |
| P01834 | *Immunoglobulin kappa constant* | IGKC | + | 75 |
| P01857 | Immunoglobulin heavy constant gamma 1 | IGHG1 | + | 82 |
| P01859 | *Immunoglobulin heavy constant gamma 2* | IGHG2 | + | 68 |
| P01876 | Immunoglobulin heavy constant alpha 1 | IGHA1 | + | 63 |
| P02489 | Alpha-crystallin A chain | CRYAA | + | 56 |
| P02511 | Alpha-crystallin B chain | CRYAB | + | 57 |
| **P02538** | **Keratin, type II cytoskeletal 6A** | KRT6A | + | 62 |
| P02647 | *Apolipoprotein A-I* | APOA1 | + | 204 |
| P02671 | *Fibrinogen alpha chain* | FGA | + | 84 |
| P02675 | *Fibrinogen beta chain* | FGB | + | 83 |
| P02679 | *Fibrinogen gamma chain* | FGG | + | 60 |
| P02750 | *Leucine-rich alpha-2-glycoprotein* | LRG | + | 60 |
| P02760 | *Alpha-1-microglycoprotein* | AMBP | + | 60 |
| P02765 | *Alpha-2-HS-glycoprotein* | AHSG | + | 60 |
| *P02766* | *Transthyretin* | *TTR* | *+* | *75* |
| P02768 | *Serum albumin* | ALB | + | 192 |
| P02787 | *Serotransferrin* | TF | - | 235 |
| P02790 | *Hemopexin* | HPX | + | 192 |
| *P04114* | *Apolipoprotein B-100* | *APOB* | *+* | *56* |
| **P04217** | **Alpha-1B-glycoprotein** | A1BG | + | 128 |
| **P04259** | **Keratin, type II cytoskeletal 6B** | KRT6B | + | 58 |
| **P04264** | **Keratin, type II cytoskeletal 1** | KRT1 | - | 81 |
| P06396 | *Gelsolin* | GSN | + | 60 |
| P06727 | *Apolipoprotein A-IV* | APOA4 | + | 60 |
| P08962 | *CD63 antigen* | CD63 | + | 60 |
| **P09601** | **Heme oxygenase 1** | HMOX1 | - | 56 |
| P0CE72 | Oncomodulin-1 | OCM | - | 56 |
| P10909 | *Clusterin* | CLU | + | 60 |
| P11310 | Medium-chain specific acyl-CoA dehydrogenase, mitochondrial | ACADM | + | 61 |
| P11586 | C-1-tetrahydrofolate synthase, cytoplasmic | MTHFD1 | + | 56 |
| P11717 | Cation-independent mannose-6-phosphate receptor | IGF2R | + | 57 |
| **P13497** | **Bone morphogenetic protein 1** | BMP1 | - | 60 |
| **P16233** | **Pancreatic triacylglycerol lipase** | PNLIP | - | 56 |
| P17540 | Creatine kinase S-type, mitochondrial | CKMT2 | - | 77 |
| P17655 | Calpain-2 catalytic subunit | CAPN2 | + | 56 |
| P18075 | Bone morphogenetic protein 7 | BMP7 | + | 56 |
| P21926 | *CD9 antigen* | CD9 | + | 60 |
| P25063 | *Signal transducer CD24* | CD24 | + | 60 |
| **P35527** | **Keratin, type I cytoskeletal 9** | KRT9 | + | 57 |
| P46779 | 60S ribosomal protein L28 | RPL28 | + | 56 |
| **P48167** | **Glycine receptor subunit beta** | GLRB | - | 56 |
| **P49761** | **Dual specificity protein kinase CLK3** | CLK3 | - | 56 |
| **P50440** | **Glycine amidinotransferase, mitochondrial** | GATM | - | 56 |
| **P55199** | **RNA polymerase II elongation factor ELL** | ELL2 | - | 56 |
| P60033 | *CD81 antigen* | CD81 | + | 60 |
| **P62987** | **Ubiquitin-60S ribosomal protein L40** | UBA52 | + | 57 |
| *P68871* | *Hemoglobin subunit beta* | *HBB* | *+* | *62* |
| P69905 | Haptoglobin alpha chain | HBA1 | + | 60 |
| **Q08426** | ***Peroxisomal bifunctional enzyme*** | EHHADH | + | 64 |
| Q13424 | *Alpha-1-syntrophin* | SNTA1 | + | 56 |
| **Q13522** | **Protein phosphatase 1A** | PPM1A | + | 56 |
| **Q13535** | **Serine/threonine-protein kinase ATR** | ATR | - | 58 |
| **Q14005** | **Pro-interleukin-16** | IL16 | - | 56 |
| **Q14320** | **Protein FAM50A** | FAM50A | - | 61 |
| **Q14571** | **Inositol 1,4,5-trisphosphate receptor type 2** | ITPR2 | + | 67 |
| **Q14624** | **Inter-alpha-trypsin inhibitor heavy chain H4** | ITIH4 | + | 80 |
| Q14966 | Zinc finger protein 638 | ZNF638 | + | 65 |
| **Q15024** | **Exosome complex component RRP42** | EXOSC7 | + | 56 |
| **Q15477** | **Helicase SKI2W** | SKIV2L | + | 65 |
| Q15776 | *Zinc finger protein with KRAB and SCAN domains 8* | ZKSCAN8 | - | 56 |
| **Q16775** | **Hydroxyacylglutathione hydrolase, mitochondrial** | HAGH | + | 56 |
| **Q16890** | **Tumor protein D53** | TPD52L1 | - | 56 |
| Q29RF7 | Sister chromatid cohesion protein PDS5 homolog A | PDS5A | + | 57 |
| **Q2M218** | **Zinc finger protein 630** | ZNF630 | - | 80 |
| **Q4G0S7** | **Coiled-coil domain-containing protein 152** | CCDC152 | - | 63 |
| **Q52M93** | **Zinc finger protein 585B** | ZNF585B | - | 57 |
| Q53FZ2 | Acyl-coenzyme A synthetase ACSM3, mitochondrial | ACSM3 | - | 59 |
| Q5CZ79 | Ankyrin repeat domain-containing protein 20B | ANKRD20A8P | - | 56 |
| Q5M9N0 | Coiled-coil domain-containing protein 158 | CCDC158 | + | 71 |
| **Q5R372** | **Rab GTPase-activating protein 1-like** | RABGAP1L | - | 56 |
| Q5VT06 | Centrosome-associated protein 350 | CEP350 | + | 80 |
| **Q5VWM5** | **PRAME family member 9/15** | PRAMEF9 | - | 58 |
| Q5VZ18 | SH2 domain-containing adapter protein E | SHE | - | 62 |
| **Q68J44** | **Dual specificity phosphatase DUPD1** | DUPD1 | - | 57 |
| **Q6P1J6** | **Phospholipase B1, membrane-associated** | PLB1 | - | 56 |
| **Q7L5N7** | **Lysophosphatidylcholine acyltransferase 2** | LPCAT2 | - | 56 |
| **Q7L5Y9** | **E3 ubiquitin-protein transferase MAEA** | MAEA | - | 56 |
| **Q7RTT3** | **Putative protein SSX9** | SSX9P | - | 189 |
| Q86TS9 | 39S ribosomal protein L52, mitochondrial | MRPL52 | - | 56 |
| **Q8IUS5** | **Epoxide hydrolase 4** | EPHX4 | - | 57 |
| **Q8IYA6** | **Cytoskeleton-associated protein 2-like** | CKAP2L | - | 65 |
| **Q8IYE0** | **Coiled-coil domain-containing protein 146** | CCDC146 | - | 57 |
| Q8IYI6 | Exocyst complex component 8 | EXOC8 | + | 58 |
| Q8N9H8 | Exonuclease mut-7 homolog | EXD3 | - | 57 |
| **Q8NDD1** | **Uncharacterized protein C1orf131** | C1orf131 | - | 56 |
| **Q8NEQ6** | **Steroid receptor-associated and regulated protein** | SRARP | - | 59 |
| Q8NEY8 | Periphilin-1 | PPHLN1 | - | 56 |
| Q8NFA2 | NADPH oxidase organizer 1 | NOXO1 | - | 58 |
| *Q8TES7* | *Fas-binding factor 1* | *FBF1* | *+* | *57* |
| **Q8WXR4** | **Myosin-IIIb** | MYO3B | + | 56 |
| **Q8WXS5** | **Voltage-dependent calcium channel gamma-8 subunit** | CACNG8 | - | 63 |
| Q8WXW3 | Progesterone-induced-blocking factor 1 | PIBF1 | - | 56 |
| **Q92622** | **Run domain Beclin-1-interacting and cysteine-rich domain-containing protein** | RUBCN | - | 56 |
| **Q92624** | **Amyloid protein-binding protein 2** | APPBP2 | - | 61 |
| Q969Q5 | Ras-related protein Rab-24 | RAB24 | - | 57 |
| Q969S3 | Zinc finger protein 622 | ZNF622 | - | 61 |
| Q96EY8 | Corrinoid adenosyltransferase | MMAB | - | 56 |
| Q96PX6 | *Coiled-coil domain-containing protein 85A* | CCDC85A | - | 56 |
| Q96Q89 | Kinesin-like protein KIF20B | KIF20B | - | 57 |
| Q96RL7 | Vacuolar protein sorting-associated protein 13A | VPS13A | - | 56 |
| **Q99623** | **Prohibitin-2** | PHB2 | + | 74 |
| Q9BT92 | Trichoplein keratin filament-binding protein | TCHP | - | 56 |
| Q9BVG8 | Kinesin-like protein KIFC3 | KIFC3 | + | 57 |
| Q9BYT9 | Anoctamin-3 | ANO3 | - | 49 |
| **Q9H497** | **Torsin-3A** | TOR3A | + | 63 |
| **Q9H4Q4** | **PR domain zinc finger protein 12** | PRDM12 | - | 56 |
| **Q9H6Z4** | ***Ran-binding protein 3*** | RANBP3 | + | 56 |
| **Q9H9E3** | **Conserved oligomeric Golgi complex subunit 4** | COG4 | - | 56 |
| Q9NQ34 | Transmembrane protein 9B | TMEM9B | - | 56 |
| **Q9NQG6** | **Mitochondrial dynamics protein MID51** | MIEF1 | - | 60 |
| **Q9NSD9** | **Phenylalanine-tRNA ligase beta subunit** | FARSB | + | 56 |
| Q9NWT6 | Hypoxia-inducible factor 1-alpha inhibitor | HIF1AN | - | 56 |
| Q9NYZ3 | G2 and S phase-expressed protein 1 | GTSE1 | + | 56 |
| **Q9NZJ4** | **Sacsin** | SACS | - | 56 |
| **Q9NZU7** | **Calcium-binding protein 1** | CABP1 | + | 56 |
| Q9P291 | Armadillo repeat-containing X-linked protein 1 | ARMCX1 | - | 56 |
| Q9UKW4 | Guanine nucleotide exchange factor VAV3 | VAV3 | - | 74 |
| Q9Y2W7 | Calsenilin | KCNIP3 | - | 58 |
| **Q9Y4E5** | **E3 SUMO-protein ligase ZNF451** | ZNF451 | + | 69 |
| Q9Y4J8 | Dystrobrevin alpha | DTNA | - | 66 |

* - Universal proteins are in italics, proteins unique to the total blood fraction are in bold.
