# Supplementary figures and images for: Proteomic Analysis of Blood Exosomes from Healthy Females and Breast Cancer Patients Reveals an Association between Different Exosomal Bioactivity on Non-tumorigenic Epithelial Cell and Breast Cancer Cell Migration in Vitro
Source: Biomolecules. 2020 Mar 25;10(4):495. doi: 10.3390/biom10040495 (PMC7226042; doi:10.3390/biom10040495)

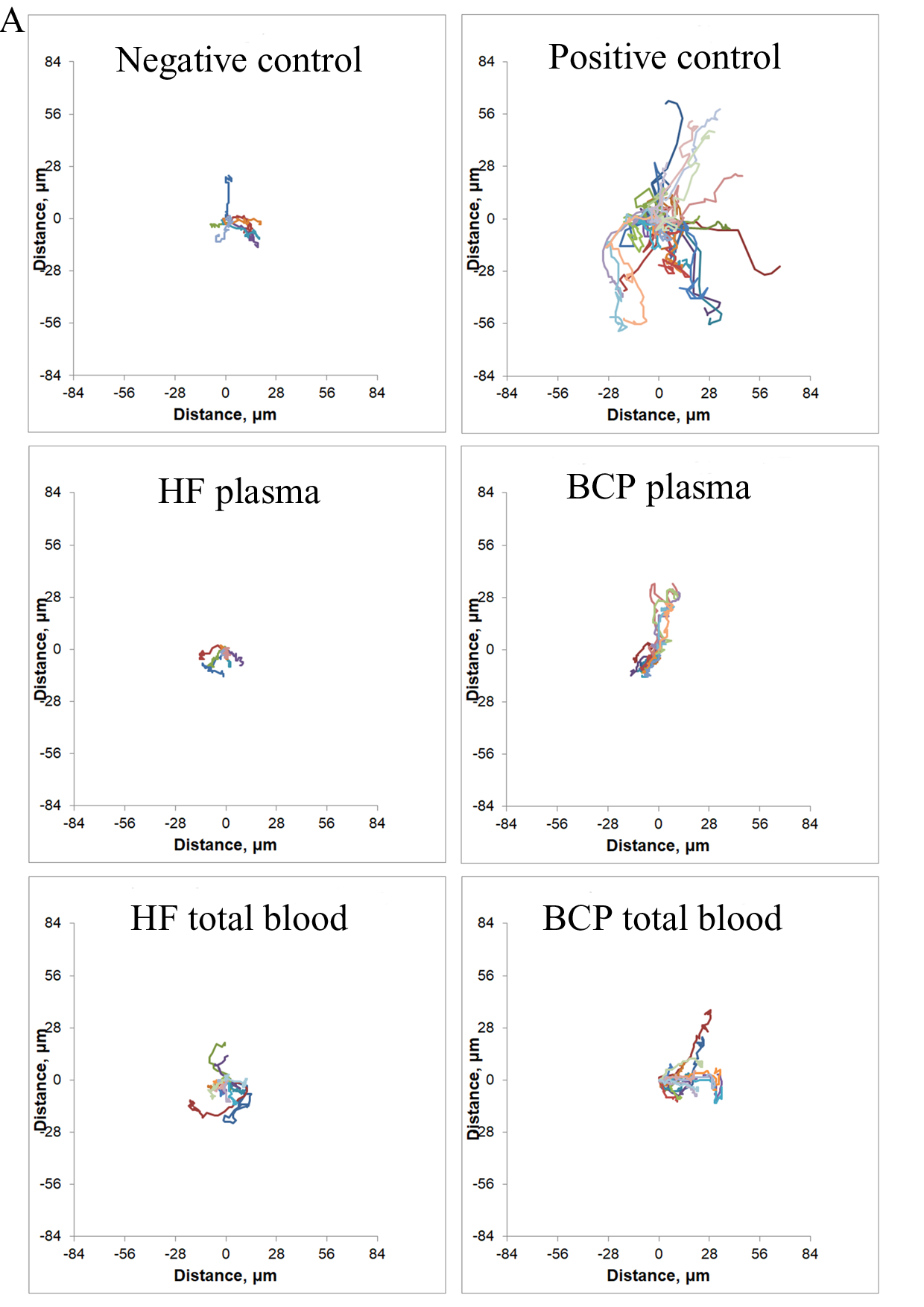

Supplement: Supplementary file 1 [file biomolecules-10-00495-s001.zip › suppl files/Suppl fig 1A.tif]

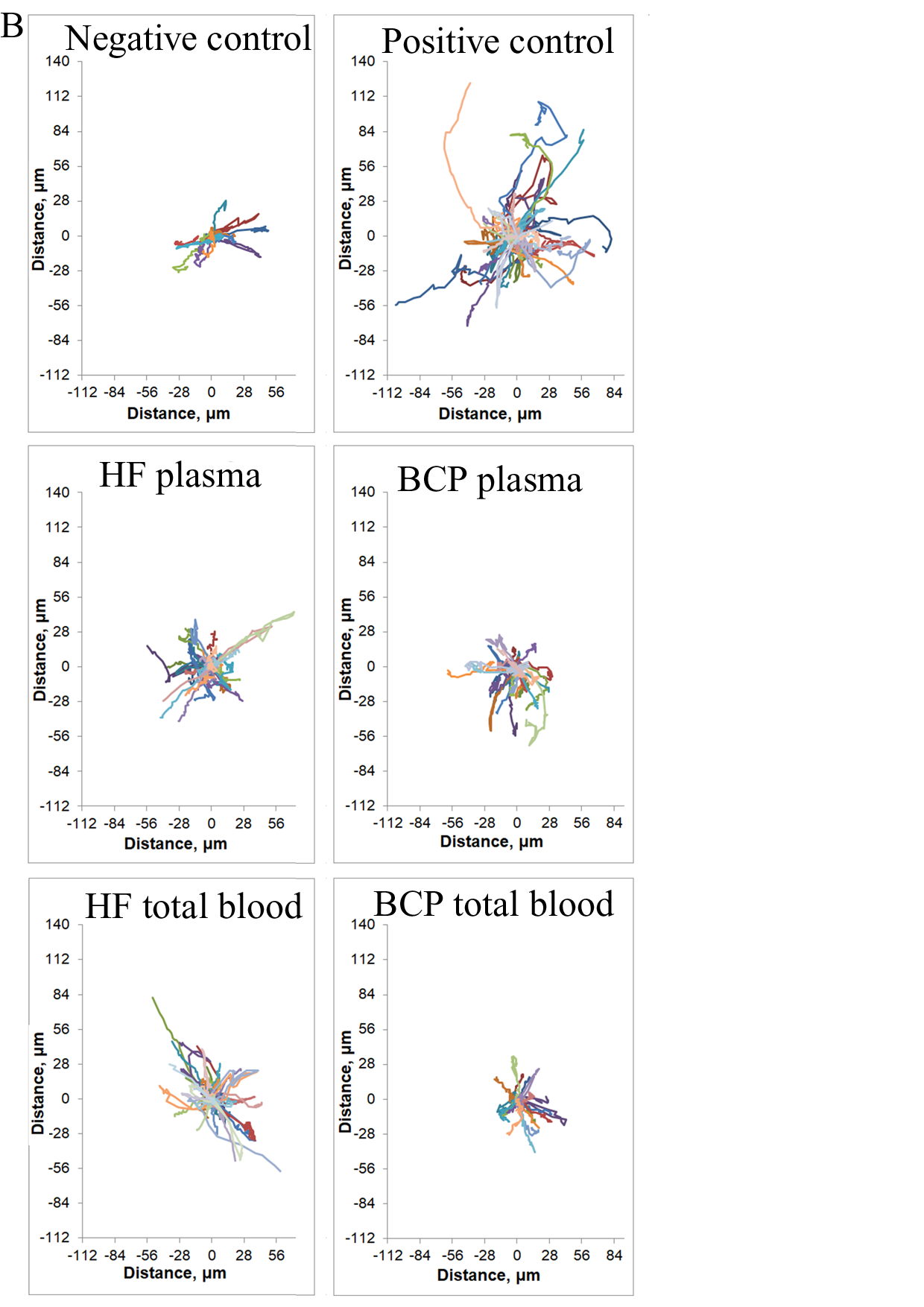

Supplement: Supplementary file 1 [file biomolecules-10-00495-s001.zip › suppl files/Suppl fig 1B.tif]

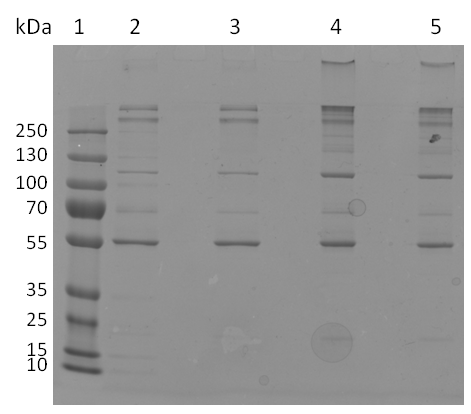

Supplement: Supplementary file 1 [file biomolecules-10-00495-s001.zip › suppl files/Suppl fig 2.tif]
